# Biocontrol Mechanisms of *Trichoderma koningiopsis* PSU3-2 against Postharvest Anthracnose of Chili Pepper

**DOI:** 10.3390/jof7040276

**Published:** 2021-04-07

**Authors:** On-Uma Ruangwong, Chaninun Pornsuriya, Kitsada Pitija, Anurag Sunpapao

**Affiliations:** 1 Department of Entomology and Plant Pathology, Faculty of Agriculture, Chiang Mai University, Mueang, Chiang Mai 50200, Thailand; on-uma.r@cmu.ac.th; 2 Innovative Agriculture Research Center, Faculty of Agriculture, Chiang Mai University, Chiang Mai 50200, Thailand; 3 Agricultural Innovation and Management Division (Pest Management), Faculty of Natural Resources, Prince of Songkla University, Hatyai, Songkhla 90110, Thailand; chaninun.p@psu.ac.th; 4 Perkin Elmer Co. Ltd., 290 Soi 17, Rama 9 Rd., Bangkapi, Huay Kwang, Bangkok 10310, Thailand; Kitsada.pitija@perkinelmer.com

**Keywords:** in vitro tests, *β*-1,3-glucanase, chitinase, electron microscopy, GC/MS profiling

## Abstract

Several mechanisms are involved in the biological control of plant pathogens by the soil-borne *Trichoderma* spp. fungi. The aim of this study was to characterize a new strain of *Trichoderma* as a potential biological control agent to control the postharvest anthracnose of chili pepper caused by *Colletotrichum* *gloeosporioides*. A total of nine strains of *Trichoderma* spp. were screened for their antifungal activity using a dual culture assay against *C.* *gloeosporioides*. *Trichoderma koningiopsis* PSU3-2 was shown to be the most effective strain, with a percentage inhibition of 79.57%, which was significantly higher than that of other strains (*p* < 0.05). In the sealed plate method, *T. koningiopsis* PSU3-2 suppressed the growth of *C.* *gloeosporioides* by 38.33%. Solid-phase microextraction (SPME) was applied to trap volatiles emitted by *T. koningiopsis* PSU3-2, and the GC/MS profiling revealed the presence of antifungal compounds including azetidine, 2-phenylethanol, and ethyl hexadecanoate. The production of cell-wall-degrading enzymes (CWDEs) was assayed through cell-free culture filtrate (CF) of PSU3-2, and the enzyme activity of chitinase and *β*-1,3-glucanase was 0.06 and 0.23 U/mL, respectively, significantly higher than that in the control (*p* < 0.05). Scanning electron microscopy of the mycelium incubated in cell-free CF of *T. koningiopsis* PSU3-2 showed the abnormal shape of *C.* *gloeosporioides* hyphae. Application of *T. koningiopsis* PSU3-2 by the dipping method significantly reduced the lesion size (*p* < 0.05) after inoculation with *C.* *gloeosporioides* compared to the control, and there was no disease symptom development in *T. koningiopsis* PSU3-2-treated chili pepper. This study demonstrates that *T. koningiopsis* PSU3-2 is an effective antagonistic microorganism and a promising biocontrol agent against postharvest anthracnose of chili pepper, acting with multiple mechanisms.

## 1. Introduction

Rhizosphere soil has long been considered as the main source of isolation of useful beneficial microorganisms [1,2]. At present, numerous soil fungi isolated from soil are employed as biological control agents, especially fungi in the genus *Trichoderma*. *Trichoderma* species are widely used to control numerous plant pathogens and reduce disease severity [3,4], due to their capacity for nutrient and space competition [5,6], parasitism [7], secretion of antimicrobial metabolites [7,8,9,10], activation of defense responses [11,12], and promotion of plant growth [8,9,13]. Moreover, metabolites, such as volatile organic compounds (VOCs), secreted from the *Trichoderma* species have been applied to promote plant growth [8,9,14]. Application of the *Trichoderma* species has been used to reduce the disease severity of leaf spots on lettuce [12] and sugar beet [15], as well as brown spots on rice [16]. Biological control presents low human health risks, as well as an environmentally friendly method without the excessive use of chemical fungicides in various crops.

Anthracnose is a common plant disease characterized by dark, sunken lesions on fruits, leaves, and stems containing conidia [17]. The causal agents of this disease, identified as *Colletotrichum* spp., reduce both the quality and the quantity of a harvest yield. Disease severity increases during the rainy season, as conidia of *Colletotrichum* are splashed and dispersed onto fresh fruit, resulting in secondary infection [18]. Anthracnose disease caused by *Colletotrichum* spp. has been reported to negatively impact the cultivation and production of mangoes [19,20], bananas [21], tomatoes [22], and chili peppers [23].

Chili anthracnose is a major constraint in chili production leading to huge losses, especially postharvest anthracnose, which causes the decay of chili pepper in tropical and subtropical regions [24,25]. Developing biological management strategies to control chili anthracnose may benefit disease management in chili peppers. This study, therefore, aimed to explore the potential of *Trichoderma* spp. isolated from soil as a biocontrol agent through dipping application. Multiple mechanisms of *Trichoderma* strains were tested for antifungal activity against *Colletotrichum gloeosporioides*.

## 2. Materials and Methods

### 2.1. Source of Trichoderma Species and Colletotrichum gloeosporioides

A total of nine *Trichoderma* strains, namely, *Trichoderma asperelloides* PSU-P1 [9], TSU1 [26], *Trichoderma asperellum* T76-14 [10], *T. koningiopsis* PSU3-2 (GenBank accession no. LC600711 and LC600712), and *Trichoderma* sp. PSU1-1, Tri1-1, Tri1-2, Tri2-1, and Tri2-2, were obtained from the Culture Collection of Pest Management (CCPM), Faculty of Natural Resources, Prince of Songkla University, whereas *Colletotrichum gloeosporioides* causing postharvest anthracnose of chili pepper was obtained from the Department of Agriculture, Ministry of Agriculture and Cooperatives, Bangkok, Thailand. *Trichoderma* and *C*. *gloeosporioides* were cultured on potato dextrose agar (PDA) (Himedia, Mumbai, India) at 28 ± 2 °C for 3 days before bioassays.

### 2.2. Dual Culture Assay

Nine strains of *Trichoderma* spp. were screened for antifungal activities on the mycelial growth of *C*. *gloeosporioides* through a dual culture assay on PDA plates [27]. An agar plug of a 5-day-old *C*. *gloeosporioides* colony was placed on the side of 9 cm Petri dishes, with an agar plug of each *Trichoderma* sp. placed on the opposite side 5 cm from the pathogen. PDA plates with pathogen alone served as the control. The experiment was designed according to a complete randomized block (CRD) with five replicates and repeated twice. The tested plates were incubated at ambient temperature (28 ± 2 °C) for 7 days. Colony radii of *C*. *gloeosporioides* were measured, and the percentage inhibition was calculated using the method of Rahman et al. [28], as given in Equation (1).

(1) $$\mathrm{Percentage}\;\mathrm{inhibition}\;(\%)=\frac{R1-R2}{R1}\times 100,$$

where R1 is the radial growth of *C*. *gloeosporioides* in control, and R2 is the radial growth of *C*. *gloeosporioides* with treatment.

### 2.3. Volatile Antifungal Bioassay and Solid-Phase Microextraction GC/MS Analysis

The effect of volatiles emitted by *Trichoderma* spp. was examined using the sealed plate method [10,29]. The most effective *Trichoderma* isolate was cultured in a 20 mL chromatography vial, 20 mm in diameter (PerkinElmer, Waltham, MA, USA), and incubated at 28 ± 2 °C for 10 days. Volatiles emitted by *Trichoderma* were trapped by solid-phase microextraction (SPME) fibers and inserted into the injection port of an SQ8 gas chromatograph (PerkinElmer, Waltham, MA, USA). GC/MS conditions adhered to the method previously described by Phoka et al. [9] and Intana et al. [10]. Total volatiles released from *Trichoderma* were tentatively identified by a computer search of the National Institute of Standards and Technology (NIST) Mass Spectral Library Search Chromatogram.

### 2.4. Liquid-Phase Cultivation and Enzyme Assay

The effective *Trichoderma* spp. were cultivated in potato dextrose broth (PDB) and incubated at 28 ± 2 °C for 5 days according to the method of Wonglom et al. [6]. The PDB-cultured *Trichoderma* spp. were filtrated with a 0.45 µm Minisart^®^ Syringe Filter (Sigma-Aldrich, St. Louis, MO, USA) and used as cell-free culture filtrate (CF). An enzyme assay was conducted to confirm that the cell-free CF of *Trichoderma* spp. contained cell-wall-degrading enzymes (CWDEs) responsible for the fungal cell-wall degradation, while chitinase and *β*-1,3-glucanase activities were assayed with 3,5-dinitrosalicylic acid (DNS), as suggested by Miller [30]. Reaction mixtures containing colloidal chitin were used as the substrate in the chitinase assay, whereas mixtures containing laminarin (Sigma-Aldrich, St. Louis, MO, USA) were used as the substrate in the *β*-1,3-glucanase assay. An assay with PDB alone served as the control. Reducing sugar released in the test reaction mixtures was measured using an ultraviolet/visible light (UV/Vis) spectrophotometer UV5300 (METASH, Shanghai, China) at 550 and 575 nm for *β*-1,3-glucanase and chitinase, respectively. Enzymes were assayed in three replicates, and the experiments were repeated twice.

### 2.5. Scanning Electron Microscopy

To test the effect of cell-free CF on fungal mycelia morphology, a scanning electron microscope (SEM) was utilized according to the method of Baiyee et al. [12]. A mycelial plug (0.5 cm) of a 7-day-old colony of *C*. *gloeosporioides* was incubated in the cell-free CF of effective *Trichoderma* strains at 37 °C for 1 h, whereas the control was incubated with PDB only. The mycelial plugs were fixed in 3% glutaraldehyde at 4 °C for 24 h and then dehydrated in a 30%, 50%, 60%, 70%, 80%, 90%, and 100% alcohol series, three times each. The samples were coated with gold and observed using a JSM-580 LV SEM (JEOL, Peabody, MA, USA) at the Science Equipment Center, Prince of Songkla University, Songkhla, Thailand.

### 2.6. In Vivo Test

A spore suspension of effective *Trichoderma* was prepared, and the concentration was adjusted with sterile distilled water (DW) to 1 × 10^6^ conidia/mL. A spore suspension of the *Colletotrichum* sp. was prepared in the same manner. Chili peppers were surface-disinfected with 70% ethanol, dipped in the spore suspension of *Trichoderma* spp., and air-dried in a laminar airflow cabinet. Chili peppers dipped in DW alone and the spore suspension of the *Colletotrichum* sp. served as the negative and positive controls, respectively. Then, 20 mL spore suspensions of *C*. *gloeosporioides* were sprayed onto the chili peppers after being dipped in the spore suspension of *Trichoderma* for 24 h and incubated in a moist box for 5 days, at which time the lesion development of all treated chili peppers was measured. Each treatment included five chili peppers (five replicates), and each experiment was repeated three times.

### 2.7. Statistical Analysis

The results regarding fungal inhibition, the enzyme assay, and lesion development were subjected to one-way analysis of variance (ANOVA). Statistically significant differences among treated samples were determined by Tukey’s test.

## 3. Results

### 3.1. Antifungal Activity of Trichoderma spp.

**Figure 1:**
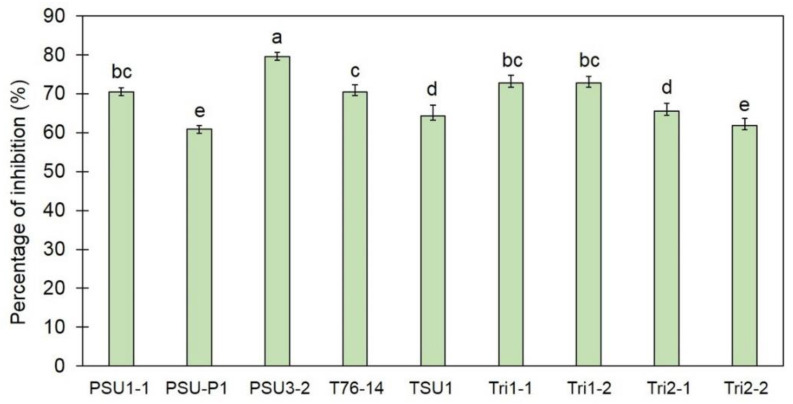
Percentage inhibition of *Trichoderma* spp. against *Colletotrichum gloeosporioides*. Different letters indicate statistically significant differences among treatments (*p* < 0.05) using Tukey’s test.

After incubation for 7 days, a smaller growth of *C*. *gloeosporioides* was observed in the dual culture plate than in the control plate. Nine strains of *Trichoderma* spp. inhibited the fungal growth of *C*. *gloeosporioides* in dual culture plates with inhibition percentages ranging from 60.84 to 79.57% (Figure 1). *T. koningiopsis* PSU3-2 was shown to be the most effective strain, with a percentage inhibition of 79.57%, statistically higher than that of other strains (*p* < 0.05) in this assay (Figure 1); therefore, the *T. koningiopsis* PSU3-2 strain was selected for further bioassays.

### 3.2. Production of Volatile Antifungal Compounds

**Figure 2:**
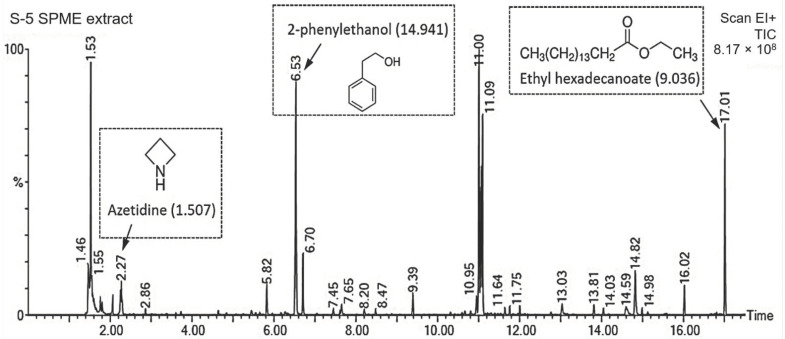
Total ion chromatogram of volatile compounds identified from *T. koningiopsis* PSU3-2 through GC/MS analysis. Peaks at 2.27, 6.53, and 17.01 min were tentatively identified as azetidine, 2-phenylethanol, and ethyl hexadecanoate, the structures of which are shown. Numbers in parentheses indicate the percentage of peak areas.

The sealed plate method showed that *T. koningiopsis* PSU3-2 inhibited the fungal growth of *C*. *gloeosporioides*, with a percentage inhibition of 38.33%. This result reveals that *T. koningiopsis* PSU3-2 produced volatile organic compounds which were responsible for suppressing the mycelial growth of *C*. *gloeosporioides* in vitro. A total of 16 volatile compounds were detected in *T. koningiopsis* PSU3-2 through GC/MS analysis. The volatile compounds contained carbon numbers ranging from C1 (fluoro(trinitro)methane) to C20 (ethyl (*E*)-octadec-9-enoate). The major compounds found in this study were 2-phenylethanol followed by fluoroethane and 1-oxacyclotetradeca-4,11-diyne, with percentage peak areas of 14.94, 12.85, and 11.588%, respectively (Table 1). According to previous literature reviews, only three compounds were reported as volatile antifungal compounds (VOCs), namely, azetidine (1.507% peak area), 2-phenylethanol (14.941%), and ethyl hexadecanoate (9.036%). Figure 2 shows the mass spectrum of volatile antifungal compounds and their structures. No major peaks were observed in PDA alone, which served as the control group.

### 3.3. Cell-Wall-Degrading Enzyme Activities

**Figure 3:**
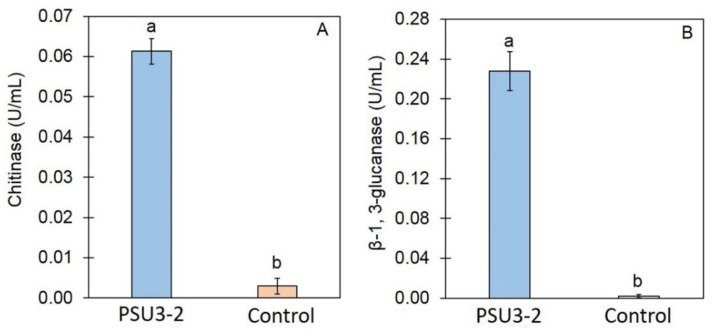
Cell-wall-degrading enzyme activities of cell-free culture filtrate (CF) of *T. koningiopsis* PSU3-2: (**A**) enzyme activity of *β*-1,3-glucanase; (**B**) enzyme activity of chitinase. Different letters indicate statistically significant differences among treatments (*p* < 0.05) using Tukey’s test.

The activity of CWDEs, including chitinase and *β*-1,3-glucanase, was assayed through the cell-free CF of *T. koningiopsis* PSU3-2. The enzyme activity of chitinase and *β*-1,3-glucanase in the cell-free CF of *T. koningiopsis* PSU3-2 was 0.061 and 0.227 U/mL (Figure 3), respectively, significantly higher (*p* < 0.05) than that in the control (PDB alone).

### 3.4. Effect of Cell-Free CF on Fungal Mycelia

**Figure 4:**
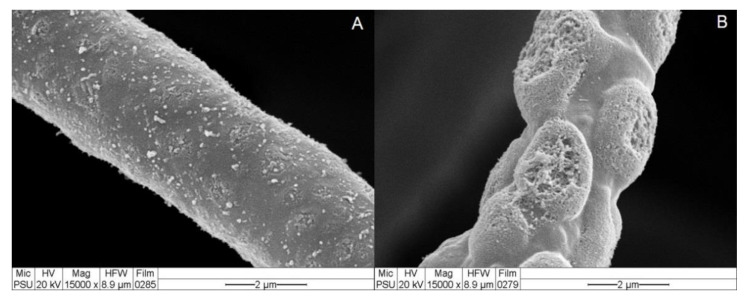
Effects of cell-wall-degrading enzymes on the fungal morphology of *C*. *gloeosporioides* (**A**) hypha of *C*. *gloeosporioides* incubated in potato dextrose broth alone; (**B**) hypha of *C*. *gloeosporioides* incubated in cell-free culture filtrate (CF) of *T. koningiopsis* PSU3-2.

SEM analysis was conducted to confirm the nature of the cell-free CF of *T. koningiopsis* PSU3-2 containing CWDEs or antifungal compounds responsible for inhibiting the fungal growth of *C*. *gloeosporioides*. The SEM micrograph of the control (PDB alone) exhibited no morphological change in the fungal mycelia of the *Colletotrichum* sp. (Figure 4), whereas the fungal mycelia incubated in the cell-free CF of *T. koningiopsis* PSU3-2 displayed abnormal shapes and mycelial distortions (Figure 4).

### 3.5. Effect of Trichoderma on Lesion Development

**Figure 5:**
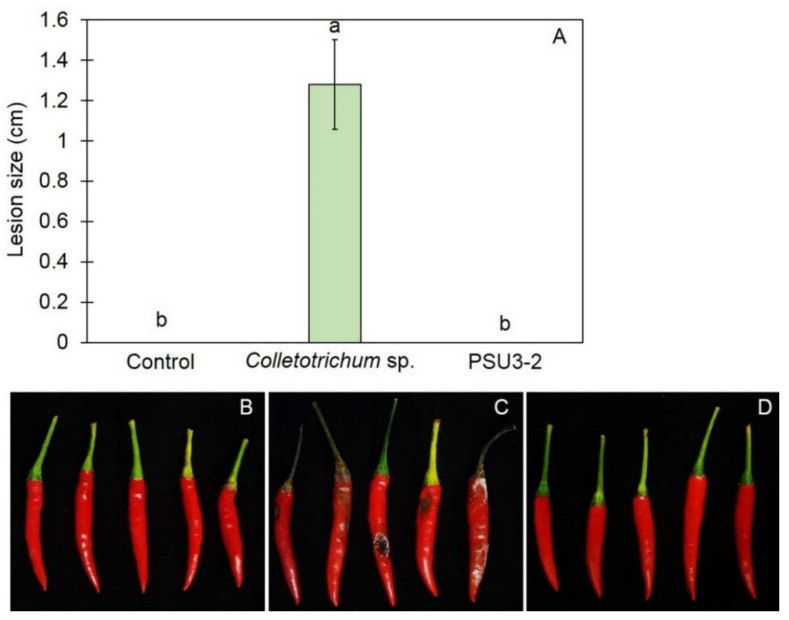
(**A**) Lesion sizes developed after inoculation with *Colletorichum* sp.; (**B**) chili pepper fruit inoculated with distilled water alone; (**C**) chili pepper fruit inoculated with *Colletorichum* sp. alone; (**D**) chili pepper fruit challenge inoculation with *T. koningiopsis* PSU3-2 and *Colletorichum* sp. Different letters indicate statistically significant differences among treatments (*p* < 0.05) using Tukey’s test.

Treatment of *T. koningiopsis* PSU3-2 using the dipping method prior to inoculation with *Colletotrichum* sp. significantly reduced the size of anthracnose lesions (*p* < 0.05) analyzed for all chili peppers in all treatments. The lesion sizes developed on the chili pepper of the untreated control group, the *Trichoderma* PSU3-2-treated chili pepper, and *C*. *gloeosporioides* inoculation alone (control) were 0, 0, and 1.28 cm in diameter, respectively (Figure 5). There was no disease development in the *T. koningiopsis* PSU3-2-treated chili pepper fruit after incubation for 5 days.

## 4. Discussion

Postharvest anthracnose of chili pepper is reportedly caused by *Colletotrichum* spp., leading to a reduction in both the quality and the quantity of chili pepper production [24,25]. This study investigated the antifungal activity of *Trichoderma* spp. against postharvest anthracnose of chili pepper fruit. *T. koningiopsis* PSU3-2 effectively suppressed the fungal growth of the *C*. *gloeosporioides*, revealing a competition mechanism (Figure 1). This isolate was documented as being capable of emitting VOCs to restrict the mycelial growth of the *C*. *gloeosporioides* (Figure 3), along with overproduction of CWDEs leading to a morphological change in the *C*. *gloeosporioides* (Figure 4). Furthermore, treatment with *T. koningiopsis* PSU3-2 protected chili peppers from postharvest anthracnose decay (Figure 5).

The ability to compete for nutrients and space is commonly found in several *Trichoderma* spp. to overcome the growth of fungal pathogens through a dual culture assay [3,4,6,31]. In vitro studies revealed the competition mechanism of *Trichoderma* spp. against *Sclerotium sclerotiorum* [32], *Rhizoctonia solani*, *Macrophomina phaseolina* [33], and *Curvularia oryzae* [3]. Our findings in this study are in agreement with previous publications that found that *T. koningiopsis* PSU3-2 grew faster than the *C*. *gloeosporioides*, effectively inhibiting the growth of the *C*. *gloeosporioides* in PDA-assayed plates, thereby suggesting a competition mechanism involved in biocontrol activity (Figure 1).

VOCs have been reported as being produced and released by several *Trichoderma* species with a diversity of volatile compounds [31]. The VOCs emitted by *Trichoderma* species display multiple functions; they have antifungal properties, induce a defense response, and promote plant growth [8,9]. Among the 16 VOCs produced by *T. koningiopsis* PSU3-2, three compounds, namely, azetidine, 2-phenylethanol, and ethyl hexadecanoate, have been reported to have antimicrobial activity [34,35,36]. For instance, 2-phenylethanol emitted from *T. asperellum* T76-14 was reported to control the postharvest fruit rot of muskmelon [10]. Therefore, the VOCs of *T. koningiopsis* PSU3-2 containing azetidine, 2-phenylethanol, and ethyl hexadecanoate may be associated with the suppression of the mycelial growth of the *C*. *gloeosporioides*, suggesting the antibiosis mechanism of *T. koningiopsis* PSU3-2. Several *Trichoderma* species produce and secrete hydrolytic enzymes responsible for degrading the fungal cell wall. The main CWDEs produced by *Trichoderma* species are chitinase and β-1,3-glucanase [37]. Chitinase restricts fungal growth by degrading chitin, the major component within the fungal cell wall [38], whereas β-1,3-glucanase hydrolyzes β-glucan to oligosaccharide and glucose [39]. A combination of both enzyme activities strongly suppresses the growth of several plant fungal pathogens [4]. Our results demonstrate a high activity of CWDEs in the cell-free CF of *T. koningiopsis* PSU3-2 (Figure 3), possibly related to the inhibition of fungal growth. We confirmed through SEM analysis that the cell-free CF of *T. koningiopsis* PSU3 contained CWDEs, which caused lysis and distortion of the *C*. *gloeosporioides* hyphae (Figure 4). The ability to produce CWDEs capable of creating mycelial lysis (holes), further resulting in fungal penetration in the host fungi, suggests mycoparasitism [40]. Baiyee et al. [4] similarly observed high activities of chitinase and β-1,3-glucanase, which caused abnormal changes in the fungal mycelia. These findings may be the result of CWDEs or some type of antifungal compound released by *T. koningiopsis* PSU3-2. However, we only studied the effects of cell-free CF, and we did not observe other metabolites in this study.

The application of a *Trichoderma* spore suspension has been shown to successfully control several plant diseases [3,16,41]. Treatment with a spore suspension of *Trichoderma* *spirale* T76-1 reduced the disease severity of lettuce leaf spots caused by *Corynespora cassiicola* and *Curvularia aeria* [4]. Root dipping with a *T. asperellum* T1 spore suspension was reported to activate defense responses in lettuce against leaf spot disease [12]. Treatment with *Trichoderma* protected tomato plants from infection by *Phytophthora nicotianae* [42]. Jogaiah et al. [43] demonstrated that the application of a *Trichoderma* *virens* spore suspension mediated resistance in tomatoes against *Fusarium* wilt by activating the jasmonic and salicylic pathways. Our study showed that chili peppers dipped in a spore suspension of *T. koningiopsis* PSU3-2 displayed no anthracnose lesions (Figure 5). Therefore, the biological activity of *T. koningiopsis* PSU3-2 is able to limit fungal infections, thereby controlling postharvest anthracnose of chili pepper fruit.

## 5. Conclusions

This study revealed the potential of a new strain of *T*. *koningiopsis* PSU3-2 isolated from soil as a biocontrol agent against anthracnose of chili pepper fruit caused by a *C*. *gloeosporioides*. The ability to compete for nutrients and space (competition), the production of VOCs (antibiosis), and the production of CWDEs (mycoparasitism) were the main factors contributing to its success in controlling the postharvest anthracnose of chili pepper fruit. The potential to develop a biopesticide to control chili anthracnose using *T*. *koningiopsis* PSU3-2 needs to be verified in the near future.

## Figures and Tables

**Table 1:** International Union of Pure and Applied Chemistry (IUPAC) names of volatile compounds produced by *T. koningiopsis* PSU3-2 identified through solid-phase microextraction (SPME)/GC/MS analysis.

Retention Time	IUPAC Name	Percentage Match	Percentage Area	Formula
1.463	fluoro(trinitro)methane	95	4.2	CFN_3_O_6_
1.528	fluoroethane	78.9	12.851	C_2_H_5_F
2.274	azetidine	89.9	1.507	C_3_H_7_N
5.824	3-isopropyl-5-methylhexan-2-one	71.8	1.581	C_10_H_20_O
6.534	2-phenylethanol	91.8	14.941	C_8_H_10_O
6.71	(4-nitrophenyl) heptanoate	79.2	3.181	C_13_H_17_NO_4_
7.65	3-methylidene-1,2-dihydroindene	88.2	0.541	C_10_H_10_
9.389	(*E*)-2,5,6-trimethylhept-4-en-3-one	74.9	1.096	C_10_H_18_O
10.95	1-oxacyclotetradeca-4,11-diyne	75.2	0.976	C_13_H_18_O
11	1-oxacyclotetradeca-4,11-diyne	76.7	11.588	C_13_H_18_O
11.09	1-oxacyclotetradeca-4,11-diyne	77.4	7.882	C_13_H_18_O
11.75	2,4-di-*tert*-butylphenol	77.4	0.41	C_14_H_22_O
13.03	cyclohex-2-en-1-ylmethylbenzene	70.5	0.809	C_13_H_16_
13.81	2,2-dimethyl-3-(3-methylpenta-2,4-dienyl)oxirane	80	0.53	C_10_H_16_O
14.59	(9*E*,12*E*)-octadeca-9,12-dienoic acid	80.2	1.131	C_18_H_32_O_2_
14.82	ethyl (*E*)-octadec-9-enoate	81.5	3.631	C_20_H_38_O_2_
16.02	ethyl pentadecanoate	83.2	1.452	C_17_H_34_O_2_
17.01	ethyl hexadecanoate	85.9	9.036	C_18_H_36_O_2_

## Data Availability

Not applicable.
